# Supplementary material for: Enterococcus faecalis redox metabolism activates the unfolded protein response to impair wound healing
Source: Sci Adv. 2026 Jan 16;12(3):eaeb5297. doi: 10.1126/sciadv.aeb5297 (PMC12810646; doi:10.1126/sciadv.aeb5297)
Supplement: Supplementary file 1 — Supplementary Methods Figs. S1 to S4 Tables S3 to S7 Legends for tables S1 and S2 Legends for movies S1 to S7 References [file sciadv.aeb5297_sm.pdf]

Science Advances
AAAS

Supplementary Materials for

Enterococcus faecalis redox metabolism activates the unfolded protein response to impair wound healing

Aaron Ming Zhi Tan *et al.*

Corresponding author: Guillaume Thibault, thibault@ntu.edu.sg; Kimberly A. Kline, kimberly.kline@unige.ch

Sci. Adv. **12**, eaeb5297 (2026)
DOI: 10.1126/sciadv.aeb5297

The PDF file includes:

Supplementary Methods
Figs. S1 to S4
Tables S3 to S7
Legends for tables S1 and S2
Legends for movies S1 to S7
References

Other Supplementary Material for this manuscript includes the following:

Tables S1 and S2
Movies S1 to S7

SUPPLEMENTAL METHODS

Ferric reductase assay

Ferric reductase activity on insoluble ferric chloride was assessed using the ferrozine assay. Overnight bacteria cultures were inoculated in tryptone soya broth (TSB, Oxoid #CM0129B), grown to mid-log phase and normalised to an OD_{600} of 0.5 in TSB supplemented with 4 g/L glucose (Sigma-Aldrich #G8270) and 0.5 mM $FeCl_3$ (Sigma-Aldrich #157740). 1 ml of this normalised culture was transferred into a microcentrifuge tube. 100 µl of sterile mineral oil was then added into each tube before they were incubated at 37°C for 1 hour. After incubation, the tubes were centrifuged at 8000 *x g* for 5 minutes. 200 µl of supernatant was transferred into a cuvette containing 800 µl of 2 mM ferrozine in PBS. The samples were then incubated in a dark environment at room temperature for 5 minutes before their OD_{562} were measured in a spectrophotometer. Iron concentration in the supernatant was calculated based on a standard curve prepared using $FeSO_4$ (Sigma-Aldrich #307718) and 10 mM L-cysteine HCl (Sigma #C121800) in PBS.

SUPPLEMENTAL FIGURES

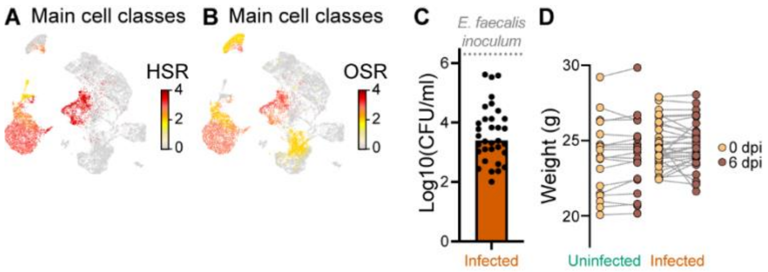

Figure S1. Stress response analysis and characterisation of the *E. faecalis* wound infection model. Related to Figure 1.

(A) Per-cell enrichment score for a curated heat shock response (HSR) gene set projected onto the uniform manifold approximation and projection (UMAP) in Fig. 1A. (**B**) Per-cell enrichment score for a curated oxidative stress response (OSR) gene set projected onto the UMAP in Fig. 1A. **(C)** Mouse wounds were inoculated with $2x10^6$ colony forming units (CFU) of *E. faecalis* WT. The CFU were quantified at 6 days post-infection (dpi) to quantify *E. faecalis* burden at the wound site (*n* = 33). **(D)** Weight of uninfected and WT-infected 6–7-week-old C57BL/6J mouse skin wounds from the day of wounding (0 dpi) until 6 dpi (uninfected, *n* = 20; WT, *n* = 33).

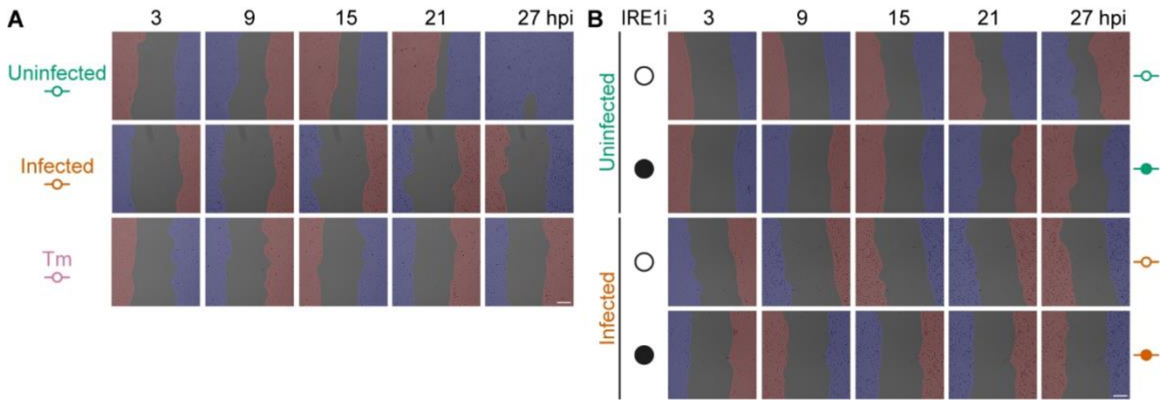

Figure S2. *In vitro* epithelial cell migration following infection and UPR modulation. Related to Figure 2.

(A-B) Representative scratch wound images for (A) uninfected, infected and Tm-treated cells, which were also (B) treated with 0.5% DMSO control (open circles) or IRE1i (close circles). Scale bars, 200 µm. Areas highlighted in red and blue demarcate the cell monolayer identified by Cellprofiler.

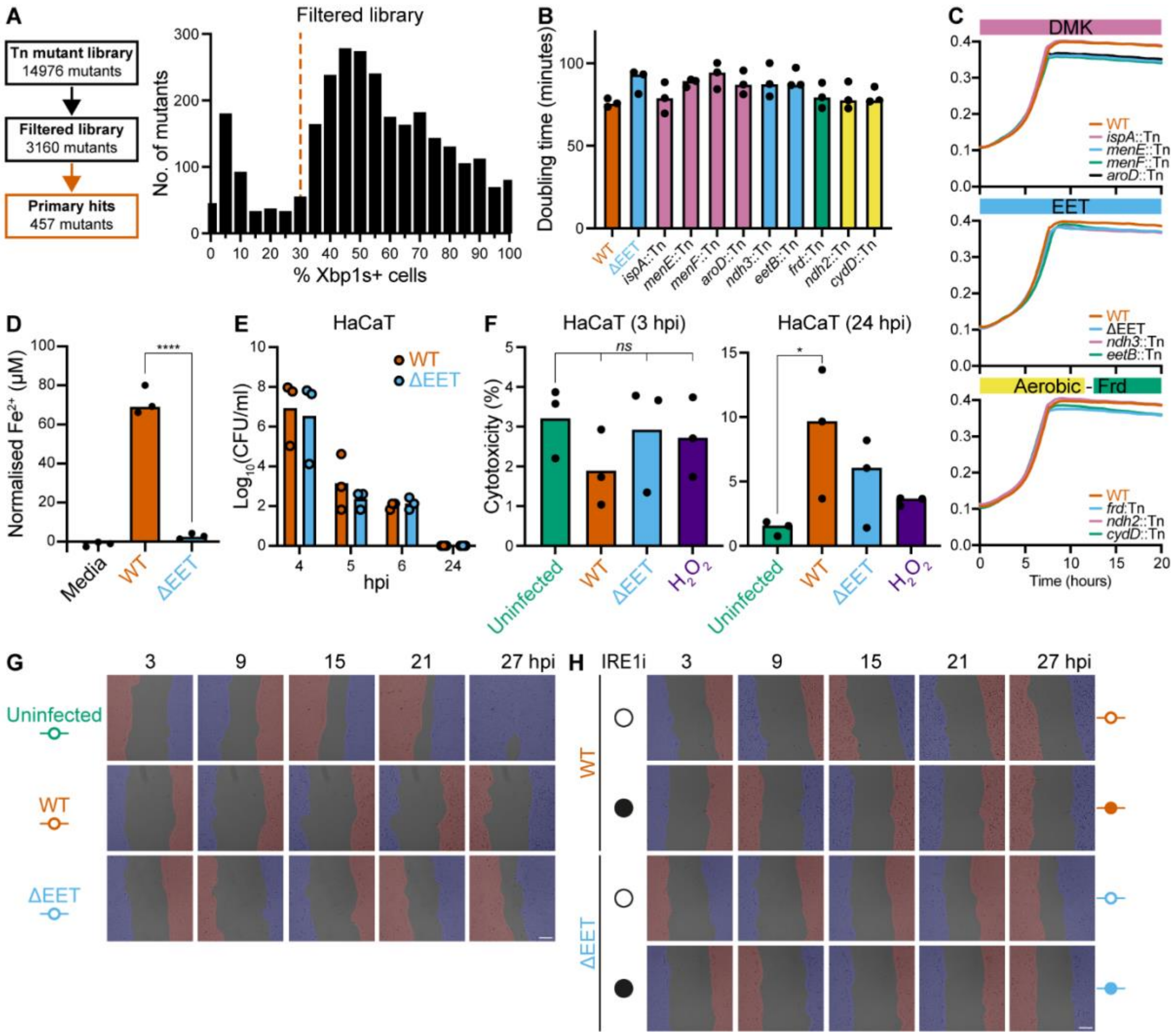

Figure S3. Selection and characterisation of *E. faecalis* mutants that fail to induce the host UPR. Related to Figure 3.

(A) Filtering and thresholding (orange dotted line) the transposon mutant library to identify primary hits for further validation. Mutants were filtered using the following criteria: (i) Contains only one transposon insertion in coding sequence, (ii) > 10 H3342+ cells in an image, (iii) > 0 Xbp1s+ cells in an image (iv) Overnight culture with an absorbance OD_{600} > 0.3. **(B-C)** Doubling times (B) and growth curves (C) of WT and UPR-defective mutants, determined by monitoring absorbance (OD_{600}) during growth in cell culture medium at 37°C. **(D)** Ferric reduction by WT and ΔEET as assessed by ferrozine assay (n = 3). **(E)** Survival of extracellular WT and ΔEET during antibiotic exposure in HaCaT co-cultures (n = 3). **(F)** Cytotoxicity at 3 and 24 hpi after exposing HaCaT to WT, ΔEET, or 250 µM H_2O_2 using a modified trypan blue assay (n = 3). **(F)** Cytotoxicity at 3 and 24 hpi after exposing HaCaT to WT, ΔEET, or 250 µM H_2O_2 using a modified trypan blue assay (n = 3).**(G-H)** Representative scratch wound images for (G) uninfected, WT-infected and ΔEET-infected cells, which were also (H) treated with 0.5% DMSO control (open circles) or IRE1i (close circles). Significance was determined using one-way ANOVA Dunnett's test (B, D, F) or two-way ANOVA Šidák test (E) (*ns*, non-significant; *p < 0.05, ****p < 0.0001). Scale bars; 200 µm. Areas highlighted in red and blue demarcate the cell monolayer identified by Cellprofiler.

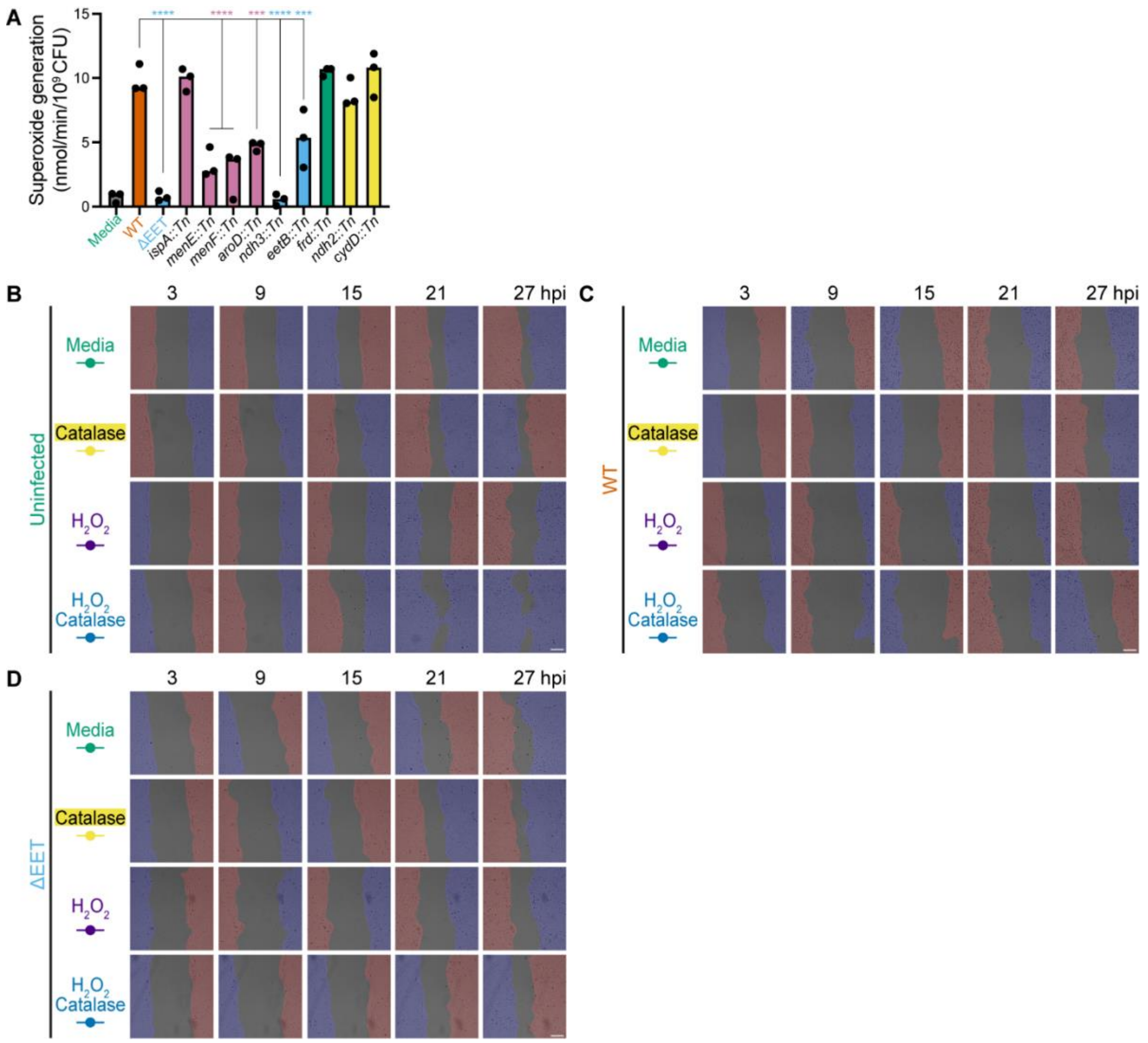

Figure S4. Representative scratch wound images in uninfected and WT-infected conditions, when treated with catalase and/or hydrogen peroxide. Related to Figure 4. (A) Superoxide generation rate of UPR defective mutants. Significance was determined using one-way ANOVA Dunnett's test (*n* = 3, ****p* < 0 .001, *****p* < 0.0001). **(B-D)** Representative scratch wound images in uninfected (B), WT-infected (C), or ΔEET-infected (D) HaCaT cells treated with catalase and/or H_2O_2. Scale bars, 200 µm. Areas highlighted in red and blue demarcate the cell monolayer identified by Cellprofiler.

SUPPLEMENTAL TABLES

Table S3. Bacterial strains. Related to Figure 1-4.

Strain	Description	Reference or source
E. faecalis		
OG1RF	Laboratory strain, Rif^R, Fus^R	Dunny et al.(84)
ΔEET	EET operon chromosomal deletion mutant, Rif^R, Fus^R	This study
Δ*ndh3*	*ndh3* chromosomal deletion mutant, Rif^R, Fus^R	This study
Δ*eetB*	*eetB* chromosomal deletion mutant, Rif^R, Fus^R	This study
ΔMEN	MEN operon chromosomal deletion mutant, Rif^R, Fus^R	This study
Δ*menE*	*menE* chromosomal deletion mutant, Rif^R, Fus^R	This study
Δ*menF*	*menF* chromosomal deletion mutant, Rif^R, Fus^R	This study
ΔT7SS	T7SS operon chromosomal deletion mutant, Rif^R, Fus^R, Cm^R	This study
ispA::*tn*	*ispA* transposon insertion mutant (Tn insertion at 740028), Rif^R, Fus^R, Cm^R	Kristich *et al.* (2008) (82)
menE::*tn*	*menE* transposon insertion mutant (Tn insertion at 344801), Rif^R, Fus^R, Cm^R	Kristich *et al.* (2008) (82)
menF::*tn*	*menF* transposon insertion mutant (Tn insertion at 345749), Rif^R, Fus^R, Cm^R	Kristich *et al.* (2008) (82)
aroD::*tn*	*aroD* transposon insertion mutant (Tn insertion at 1501457), Rif^R, Fus^R, Cm^R	Kristich *et al.* (2008) (82)
ndh3::*tn*	*ndh3* transposon insertion mutant (Tn insertion at 2658965), Rif^R, Fus^R, Cm^R	Kristich *et al.* (2008) (82)
eetB::*tn*	*eetB* transposon insertion mutant (Tn insertion at 2660397), Rif^R, Fus^R, Cm^R	Kristich *et al.* (2008) (82)
frd::*tn*	*frd* transposon insertion mutant (Tn insertion at 2043345), Rif^R, Fus^R, Cm^R	Kristich *et al.* (2008) (82)
ndh2::*tn*	*ndh2* transposon insertion mutant (Tn insertion at 1729431), Rif^R, Fus^R, Cm^R	Kristich *et al.* (2008) (82)
cydD::*tn*	*cydD* transposon insertion mutant (Tn insertion at 1735842), Rif^R, Fus^R, Cm^R	Kristich *et al.* (2008) (82)
E. coli		
Stellar	*E. coli* host strain for routine cloning	Laboratory stock
DH5a	*E. coli* host strain for routine cloning	Laboratory stock

Table S4. Oligonucleotide primers used in this study. Related to Figure 1-4.

Species	*Gene*	Forward (5' to 3')	Reverse (5' to 3')
Human	*CHOP*	GGA AAC AGA GTG GTC ATT CCC	CTG CTT GAG CCG TTC ATT CTC
	EDEM1	ACG AGC AGT GAA AGC CCT TTG G	CCA CTC TGC TTT CCA ACC CAG T
	GAPDH	GCC ATC AAT GAC CCC TTC ATT	TCT CGC TCC TGG AAG ATG G
	HERPUD1	TCG TGG TTC TAA TCG GGG ACA	CCA GGG GAA GAA AGG TTC CG
	XBP1s	TGC TGA GTC CGC AGC AGG TG	GCT GGC AGG CTC TGG GGA AG
Mouse	*Chop*	AAG ATG AGC GGG TGG CAG CG	GCT CCC AGC TGG ACA CCG TC
	Gapdh	TCA GGA GAG TGT TTC CTC GTC CC	TCT CGG CCT TGA CTG TGC CG
	Herpud1	TTG GAG CTG AGT GGC GAC CG	GGA AGC AAA TCT TGG AGA CAC TGG T
Omni (85)	*XBP1s*	GCT GAG TCC GCA GCA GGT	CWG GGT CCA ACT TGW MCA GAA T
	GAPDH	ACC ATC TTC CAG GAG CGA GA	GGG CCA TCC ACA GTC TTC TG

Table S5. Antibodies used in this study. Related to Figure 2.

Target	Host	Dilution for IB	Reference or source
XBP1s/XBP1u	Rabbit	1:500	Cell signalling technology #12782
BiP	Rabbit	1:1,000	Cell signalling technology #3177
α-TUBULIN	Mouse	1:10,000	DSHB #12G10
IRDye 800CW Goat anti-Rabbit IgG	Goat	1:15,000	Licor #926-32211
IRDye 680LT Goat anti-Mouse IgG	Goat	1:15,000	Licor # 926-68020

Table S6. Plasmids used in this study. Related to Figure 3-4.

Plasmid	Relevant characteristics	Reference or source
pLVX-Xbp1s-mApple	Lentiviral transfer plasmid for XBP1 splicing reporter	This study
pLP1	Lentiviral packaging plasmid	Thermo Fisher #K497500
pLP2	Lentiviral packaging plasmid	Thermo Fisher #K497500
pLP/VSVG	Lentiviral packaging plasmid	Thermo Fisher #K497500
pCGP213	Thermosensitive shuttle plasmid	Nielsen *et al.* (2012) (83)

Table S7. Primers used to generate and validate clean deletion *E. faecalis* mutants. Related to Figure 3-4.

Mutant	Primers	5' to 3'
ΔEET	Oligo P1	GCG TAA TCA TAT CGA TGG TCA TAG C
	Oligo P2	CTG GAA TTC TGC AGA TAT CCA TCA C
	Oligo P3	TCT GCA GAA TTC CAG GCC AAG CAA CGC ACT AAT
	Oligo P4	TCG ATA TGA TTA CGC ATT TTC TCT TGT CAA AAT CGT TTG T
	Screen F	CGA ACG AAC CAG AAC CAG CC
	Screen R	AAC GGG TCA GCA TTA TCG GG
	Intern F	GCG GGT GTT CTC GGA TTT TGG
	Intern R	CTT AAC GGT TCA AAC CGG CTG G
Δ*ndh3*	Oligo P1	GAT TGT CAG CAA TCG ATA ATC GAT TGA CA
	Oligo P2	GGC GGC CGT TAC TAG TGC GAC TTT TCT TAA TGA ATT CTT TGA
	Oligo P3	TAC CGA GCT CGG ATC CTC ACA AAC ATC AAA AGT TTG TGA TAC A
	Oligo P4	CGA TTG CTG ACA ATC CGC TCC TTA ATA TTG T
	Screen F	AGC AAT CGA TAA TCG ATT GAC A
	Screen R	GCG ACT TTT CTT AAT GAA TTC TTT GA
Δ*eetB*	Oligo P1	GTC ATG GAC TGC AGC TTG AA
	Oligo P2	ATT GCT ATG CTC GTT GAA CGA AAC ATA AGT GGA G
	Oligo P3	CTC CAC TTA TGT TTC GTT CAA CGA GCA TAG CAA TAT A
	Oligo P4	GGT GAT CGG ATC CGC GTG AA
	Screen F	GGT ATT CCA CAT AGG ATG TA
	Screen R	GTT TAT CAC CGT GGA AGC T
ΔMEN	Oligo P1	CAC CTA AAC TGC AGG CAG TTT C
	Oligo P2	GAC TGT ATT TTA GCA AAT TCA TTC TTT AAT TGT TGT CCA G
	Oligo P3	CTG GAC AAC AAT TAA AGA ATG AAT TTG CTA AAA TAC AGT C
	Oligo P4	CGG GAT CCC GCT ACC ACT CAT TTT AA
	Screen F	CAG TCC TTT CTA ATA AAA GAG AG
	Screen R	GTC CTA ATT GTA AAT GGT GAA
	Intern R	GCA CCA ATA ATC GTT TTA AC
Δ*menE*	Oligo P1	CAC GTT TAA ATG TTC TAG ATT TAC AAC G
	Oligo P2	GCC TCT CTT TCT TCT GTC TTG TAT AGT AAA GAA GTG
	Oligo P3	CACT TCT TTA CTA TAC AAG ACA GAA GAA AGA GAG GCG T
	Oligo P4	GAG CCC CGG TAC CAG AAA TAG
	Screen F	GAT GTG ACA ATG GAG TGG GC
	Screen R	CCT TGG TAT GCC TTT TCA CC
	Intern R	CTT TAA AGC TGG CGC TGT AG
Δ*menF*	Oligo P1	CAT GGG CTA TCT AGA TGC ACA AG
	Oligo P2	CCT CGT AAC ATC GGT AGG CTT GTC GTA ATT C
	Oligo P3	GAA TTA CGA CAA GCC TAC CGA TGT TAC GAG GAA TTG
	Oligo P4	GTT TGC AGC TGC AGT TCC AGA G
	Screen F	CAA GAC CAG TGT TCA CGA AA
	Screen R	GAA CGT TCA TCG ACA TCG AC
	Intern R	CCT TGT CAA TCT TTG TTT CCG
ΔT7SS	Oligo P1	GCG TAA TCA TAT CGA TGG TCA TAG C
	Oligo P2	CTG GAA TTC TGC AGA TAT CCA TCA C
	Oligo P3	TCT GCA GAA TTC CAG AAA ATT TTA TCA ATT GGC AAT CA
	Oligo P4	TCG ATA TGA TTA CGC CCA ATT TTC GGT GTT CAC AGC CTG
	Screen F	GGG AAT GGC ACC CTG AAA GA
	Screen R	CTT CGC GCT TGG CTT TTT GA

Other Supplementary Materials

Table S1. Stress Response Gene Set. Related to Figure 1. Excel Spreadsheet.

Table S2. Transposon screen hits. Related to Figure 3. Excel Spreadsheet

Video S1. *E. faecalis* infection and tunicamycin treatment impair HaCaT cell migration. Related to Figure 2E.

Video S2. IRE1 inhibition alters HaCaT cell migration in uninfected and *E. faecalis*-infected conditions. Related to Figure 2G.

Video S3. The ΔEET mutant does not impair HaCaT cell migration. Related to Figure 3E.

Video S4. The effect of IRE1 inhibition on HaCaT cell migration during infection with WT or ΔEET *E. faecalis*. Related to Figure 3F.

Video S5. Catalase rescues H_2O_2-induced migration defects in uninfected HaCaT cells. Related to Figure 4K.

Video S6. Catalase restores migration in *E. faecalis*-infected HaCaT cells. Related to Figure 4L.

Video S7. Effects of catalase and hydrogen peroxide on ΔEET-infected HaCaT cells. Related to Figure 4M

REFERENCES

1. J.-H. Chng, K. K. L. Chong, L. N. Lam, J. Jie Wong, K. A. Kline, Biofilm-associated infection by enterococci. *Nat. Rev. Microbiol.* **17**, 82–94 (2019).

2. E. Fiore, D. Van Tyne, M. S. Gilmore, Pathogenicity of enterococci. *Microbiol. Spectr.* **7**, 10.1128/microbiolspec.gpp3-0053-2018 (2019).

3. K. K. L. Chong, W. H. Tay, B. Janela, A. M. H. Yong, T. H. Liew, L. Madden, D. Keogh, T. M. S. Barkham, F. Ginhoux, D. L. Becker, K. A. Kline, *Enterococcus faecalis* modulates immune activation and slows healing during wound infection. *J. Infect. Dis.* **216**, 1644–1654 (2017).

4. M. Ramsey, A. Hartke, M. Huycke, "The physiology and metabolism of enterococci," in *Enterococci: From Commensals to Leading Causes of Drug Resistant Infection,* M. S. Gilmore, D. B. Clewell, Y. Ike, N. Shankar, Eds. (Massachusetts Eye and Ear Infirmary, 2014); www.ncbi.nlm.nih.gov/books/NBK190432/.

5. X. H. Fun, G. Thibault, Lipid bilayer stress and proteotoxic stress-induced unfolded protein response deploy divergent transcriptional and non-transcriptional programmes. *Biochim. Biophys. Acta Mol. Cell Biol. Lipids* **1865**, 158449 (2020).

6. K. Halbleib, K. Pesek, R. Covino, H. F. Hofbauer, D. Wunnicke, I. Hänelt, G. Hummer, R. Ernst, Activation of the unfolded protein response by lipid bilayer stress. *Mol. Cell* **67**, 673–684.e8 (2017).

7. C. Hetz, K. Zhang, R. J. Kaufman, Mechanisms, regulation and functions of the unfolded protein response. *Nat. Rev. Mol. Cell Biol.* **21**, 421–438 (2020).

8. N. Ho, W. S. Yap, J. Xu, H. Wu, J. H. Koh, W. W. B. Goh, B. George, S. C. Chong, S. Taubert, G. Thibault, Stress sensor Ire1 deploys a divergent transcriptional program in response to lipid bilayer stress. *J. Cell Biol.* **219**, e201909165 (2020).

9. M. Baruch, I. Belotserkovsky, B. B. Hertzog, M. Ravins, E. Dov, K. S. McIver, Y. S. Le Breton, Y. Zhou, C. Y. Cheng, E. Hanski, An extracellular bacterial pathogen modulates host metabolism to regulate its own sensing and proliferation. *Cell* **156**, 97–108 (2014).

10. J. A. Smith, M. Khan, D. D. Magnani, J. S. Harms, M. Durward, G. K. Radhakrishnan, Y.-P. Liu, G. A. Splitter, Brucella induces an unfolded protein response via TcpB that supports intracellular replication in macrophages. *PLOS Pathog.* **9**, e1003785 (2013).

11. J. Celli, R. M. Tsolis, Bacteria, the ER and the unfolded protein response: Friends or foes? *Nat. Rev. Microbiol.* **13**, 71–82 (2015).

12. A. M. Keestra-Gounder, R. M. Tsolis, NOD1 and NOD2: Beyond peptidoglycan sensing. *Trends Immunol.* **38**, 758–767 (2017).

13. D. N. Bronner, B. H. Abuaita, X. Chen, K. A. Fitzgerald, G. Nuñez, Y. He, X.-M. Yin, M. X. D. O'Riordan, Endoplasmic reticulum stress activates the inflammasome via NLRP3- and caspase-2-driven mitochondrial damage. *Immunity* **43**, 451–462 (2015).

14. A. Vajjala, D. Biswas, W. H. Tay, E. Hanski, K. A. Kline, Streptolysin-induced endoplasmic reticulum stress promotes group A streptococcal host-associated biofilm formation and necrotising fasciitis. *Cell. Microbiol.* **21**, e12956 (2019).

15. J.-A. Choi, C.-H. Song, Insights into the role of endoplasmic reticulum stress in infectious diseases. *Front. Immunol.* **10**, 3147 (2019).

16. H. Tsutsuki, K. Yahiro, K. Ogura, K. Ichimura, S. Iyoda, M. Ohnishi, S. Nagasawa, K. Seto, J. Moss, M. Noda, Subtilase cytotoxin produced by locus of enterocyte effacement-negative Shiga-toxigenic *Escherichia coli* induces stress granule formation. *Cell. Microbiol.* **18**, 1024–1040 (2016).

17. E. F. A. van 't Wout, A. van Schadewijk, R. van Boxtel, L. E. Dalton, H. J. Clarke, J. Tommassen, S. J. Marciniak, P. S. Hiemstra, Virulence factors of *Pseudomonas aeruginosa* induce both the unfolded protein and integrated stress responses in airway epithelial cells. *PLOS Pathog.* **11**, e1004946 (2015).

18. M. M. Huycke, W. Joyce, M. F. Wack, Augmented production of extracellular superoxide by blood isolates of *Enterococcus faecalis*. *J. Infect. Dis.* **173**, 743–745 (1996).

19. M. M. Huycke, D. Moore, W. Joyce, P. Wise, L. Shepard, Y. Kotake, M. S. Gilmore, Extracellular superoxide production by *Enterococcus faecalis* requires demethylmenaquinone and is attenuated by functional terminal quinol oxidases. *Mol. Microbiol.* **42**, 729–740 (2001).

20. P. Pallepati, D. A. Averill-Bates, Activation of ER stress and apoptosis by hydrogen peroxide in HeLa cells: Protective role of mild heat preconditioning at 40°C. *Biochim. Biophys. Acta Mol. Cell Res.* **1813**, 1987–1999 (2011).

21. M. M. Huycke, V. Abrams, D. R. Moore, *Enterococcus faecalis* produces extracellular superoxide and hydrogen peroxide that damages colonic epithelial cell DNA. *Carcinogenesis* **23**, 529–536 (2002).

22. C. Celik, S. T. T. Lee, F. R. Tanoto, M. Veleba, K. Kline, G. Thibault, Decoding the complexity of delayed wound healing following *Enterococcus faecalis* infection. *eLife* **13**, RP95113 (2024).

23. N. Madrazo, Z. Khattar, E. T. Powers, J. D. Rosarda, R. L. Wiseman, Mapping stress-responsive signaling pathways induced by mitochondrial proteostasis perturbations. *Mol. Biol. Cell* **35**, ar74 (2024).

24. Z. Wang, P. Butler, D. Ly, M. Spiotto, A. Koong, G. Yang, Activation of the unfolded protein response in wound healing. *J. Surg. Res.* **158**, 209 (2010).

25. E. Bachar-Wikstrom, M. Manchanda, R. Bansal, M. Karlsson, P. Kelly-Pettersson, O. Sköldenberg, J. D. Wikstrom, Endoplasmic reticulum stress in human chronic wound healing: Rescue by 4-phenylbutyrate. *Int. Wound J.* **18**, 49–61 (2021).

26. L. Qiang, S. Yang, Y.-H. Cui, Y.-Y. He, Keratinocyte autophagy enables the activation of keratinocytes and fibroblasts and facilitates wound healing. *Autophagy* **17**, 2128–2143 (2021).

27. J. Yoo, E. H. Mashalidis, A. C. Y. Kuk, K. Yamamoto, B. Kaeser, S. Ichikawa, S.-Y. Lee, GlcNAc-1-P-transferase–tunicamycin complex structure reveals basis for inhibition of N-glycosylation. *Nat. Struct. Mol. Biol.* **25**, 217–224 (2018).

28. K. Mori, Signalling pathways in the unfolded protein response: Development from yeast to mammals. *J. Biochem.* **146**, 743–750 (2009).

29. C. Hetz, The unfolded protein response: Controlling cell fate decisions under ER stress and beyond. *Nat. Rev. Mol. Cell Biol.* **13**, 89–102 (2012).

30. M. Calfon, H. Zeng, F. Urano, J. H. Till, S. R. Hubbard, H. P. Harding, S. G. Clark, D. Ron, IRE1 couples endoplasmic reticulum load to secretory capacity by processing the XBP-1 mRNA. *Nature* **415**, 92–96 (2002).

31. N. Hosokawa, I. Wada, K. Hasegawa, T. Yorihuzi, L. O. Tremblay, A. Herscovics, K. Nagata, A novel ER α-mannosidase-like protein accelerates ER-associated degradation. *EMBO Rep.* **2**, 415–422 (2001).

32. B. C. Cross, P. J. Bond, P. G. Sadowski, B. K. Jha, J. Zak, J. M. Goodman, R. H. Silverman, T. A. Neubert, I. R. Baxendale, D. Ron, The molecular basis for selective inhibition of unconventional mRNA splicing by an IRE1-binding small molecule. *Proc. Natl. Acad. Sci. U.S.A.* **109**, E869–E878 (2012).

33. Y. Demay, J. Perochon, S. Szuplewski, B. Mignotte, S. Gaumer, The PERK pathway independently triggers apoptosis and a Rac1/Slpr/JNK/Dilp8 signaling favoring tissue homeostasis in a chronic ER stress *Drosophila* model. *Cell Death Dis.* **5**, e1452 (2014).

34. G. Ong, R. Ragetli, K. Mnich, B. W. Doble, W. Kammouni, S. E. Logue, IRE1 signaling increases PERK expression during chronic ER stress. *Cell Death Dis.* **15**, 276 (2024).

35. M. Baruch, B. B. Hertzog, M. Ravins, A. Anand, C. Catherine Youting, D. Biswas, B. Tirosh, E. Hanski, Induction of endoplasmic reticulum stress and unfolded protein response constitutes a pathogenic strategy of group A streptococcus. *Front. Cell. Infect. Microbiol.* **4**, 105 (2014).

36. A. Chatterjee, J. L. E. Willett, G. M. Dunny, B. A. Duerkop, Phage infection and sub-lethal antibiotic exposure mediate *Enterococcus faecalis* type VII secretion system dependent inhibition of bystander bacteria. *PLOS Genet.* **17**, e1009204 (2021).

37. L. Hederstedt, L. Gorton, G. Pankratova, Two routes for extracellular electron transfer in *Enterococcus faecalis*. *J. Bacteriol.* **202**, e00725-19 (2020).

38. N. Pierre, C. Barbé, H. Gilson, L. Deldicque, J.-M. Raymackers, M. Francaux, Activation of ER stress by hydrogen peroxide in C2C12 myotubes. *Biochem. Biophys. Res. Commun.* **450**, 459–463 (2014).

39. X. Xue, J.-H. Piao, A. Nakajima, S. Sakon-Komazawa, Y. Kojima, K. Mori, H. Yagita, K. Okumura, H. Harding, H. Nakano, Tumor necrosis factor α (TNFα) induces the unfolded protein response (UPR) in a reactive oxygen species (ROS)-dependent fashion, and the UPR counteracts ROS accumulation by TNFα. *J. Biol. Chem.* **280**, 33917–33925 (2005).

40. E. Vladykovskaya, S. D. Sithu, P. Haberzettl, N. S. Wickramasinghe, M. L. Merchant, B. G. Hill, J. McCracken, A. Agarwal, S. Dougherty, S. A. Gordon, D. A. Schuschke, O. A. Barski, T. O'Toole, S. E. D'Souza, A. Bhatnagar, S. Srivastava, Lipid peroxidation product 4-hydroxy-trans-2-nonenal causes endothelial activation by inducing endoplasmic reticulum stress. *J. Biol. Chem.* **287**, 11398–11409 (2012).

41. K. J. Simpson, L. M. Selfors, J. Bui, A. Reynolds, D. Leake, A. Khvorova, J. S. Brugge, Identification of genes that regulate epithelial cell migration using an siRNA screening approach. *Nat. Cell Biol.* **10**, 1027–1038 (2008).

42. R. Bhattacharya, F. Xu, G. Dong, S. Li, C. Tian, B. Ponugoti, D. T. Graves, Effect of bacteria on the wound healing behavior of oral epithelial cells. *PLOS ONE* **9**, e89475 (2014).

43. P. A. Swanson, A. Kumar, S. Samarin, M. Vijay-Kumar, K. Kundu, N. Murthy, J. Hansen, A. Nusrat, A. S. Neish, Enteric commensal bacteria potentiate epithelial restitution via reactive oxygen species-mediated inactivation of focal adhesion kinase phosphatases. *Proc. Natl. Acad. Sci. U.S.A.* **108**, 8803–8808 (2011).

44. S. H. Light, L. Su, R. Rivera-Lugo, J. A. Cornejo, A. Louie, A. T. Iavarone, C. M. Ajo-Franklin, D. A. Portnoy, A flavin-based extracellular electron transfer mechanism in diverse Gram-positive bacteria. *Nature* **562**, 140–144 (2018).

45. S. H. Light, R. Méheust, J. L. Ferrell, J. Cho, D. Deng, M. Agostoni, A. T. Iavarone, J. F. Banfield, S. E. F. D'Orazio, D. A. Portnoy, Extracellular electron transfer powers flavinylated extracellular reductases in Gram-positive bacteria. *Proc. Natl. Acad. Sci. U.S.A.* **116**, 26892–26899 (2019).

46. T. Ueki, Cytochromes in extracellular electron transfer in geobacter. *Appl. Environ. Microbiol.* **87**, e03109-20 (2021).

47. G. Reguera, K. D. McCarthy, T. Mehta, J. S. Nicoll, M. T. Tuominen, D. R. Lovley, Extracellular electron transfer via microbial nanowires. *Nature* **435**, 1098–1101 (2005).

48. D. Keogh, L. N. Lam, L. E. Doyle, A. Matysik, S. Pavagadhi, S. Umashankar, P. M. Low, J. L. Dale, Y. Song, S. P. Ng, C. B. Boothroyd, G. M. Dunny, S. Swarup, R. B. H. Williams, E. Marsili, K. A. Kline, Extracellular electron transfer powers enterococcus faecalis biofilm metabolism. *mBio* **9**, e00626-17 (2018).

49. F. K. Ho, L. N. Lam, A. Matysik, T. D. Watts, J. J. Wong, K. K. L. Chong, P. Y. Choo, J. Tolar, P. M. Low, Z. S. Chua, J. J. Paxman, B. Heras, E. Marsili, C. M. Ajo-Franklin, K. A. Kline, Role of sortase-assembled Ebp pili in *Enterococcus faecalis* adhesion to iron oxides and its impact on extracellular electron transfer. *Microbiol. Spectr.* **13**, e02337-24 (2025).

50. E. T. Stevens, W. Van Beeck, B. Blackburn, S. Tejedor-Sanz, A. R. M. Rasmussen, M. E. Carter, E. Mevers, C. M. Ajo-Franklin, M. L. Marco, *Lactiplantibacillus plantarum* uses ecologically relevant, exogenous quinones for extracellular electron transfer. *MBio* **14**, e02234-23 (2023).

51. C. A. Z. Tan, K. K. L. Chong, D. Y. X. Yeong, C. H. M. Ng, M. H. Ismail, Z. H. Yap, V. Khetrapal, V. S. Y. Tay, D. I. Drautz-Moses, Y. Ali, S. L. Chen, K. A. Kline, Purine and

carbohydrate availability drive *Enterococcus faecalis* fitness during wound and urinary tract infections. *MBio* **15**, e02384-23 (2023).

52. A.-L. Cabello, K. Wells, W. Peng, H.-Q. Feng, J. Wang, D. F. Meyer, C. Noroy, E.-S. Zhao, H. Zhang, X. Li, H. Chang, G. Gomez, Y. Mao, K. L. Patrick, R. O. Watson, W. K. Russell, A. Yu, J. Zhong, F. Guo, M. Li, M. Zhou, X. Qian, K. S. Kobayashi, J. Song, S. Panthee, Y. Mechref, T. A. Ficht, Q.-M. Qin, P. De Figueiredo, Brucella-driven host N-glycome remodeling controls infection. *Cell Host Microbe* **32**, 588–605.e9 (2024).

53. M. Hernandez-Morfa, N. B. Olivero, V. E. Zappia, G. E. Piñas, N. M. Reinoso-Vizcaino, M. B. Cian, M. Nuñez-Fernandez, P. R. Cortes, J. Echenique, The oxidative stress response of *Streptococcus pneumoniae*: Its contribution to both extracellular and intracellular survival. *Front. Microbiol.* **14**, 1269843 (2023).

54. T. Barichello, J. S. Generoso, L. R. Simões, S. G. Elias, J. Quevedo, Role of oxidative stress in the pathophysiology of pneumococcal meningitis. *Oxid. Med. Cell. Longev.* **2013**, 371465 (2013).

55. G. W. Lau, H. Ran, F. Kong, D. J. Hassett, D. Mavrodi, *Pseudomonas aeruginosa* pyocyanin is critical for lung infection in mice. *Infect. Immun.* **72**, 4275–4278 (2004).

56. S. Sinha, X. Shen, F. Gallazzi, Q. Li, J. W. Zmijewski, J. R. Lancaster, K. S. Gates, Generation of reactive oxygen species mediated by 1-hydroxyphenazine, a virulence factor of *Pseudomonas aeruginosa*. *Chem. Res. Toxicol.* **28**, 175–181 (2015).

57. S. J. Dixon, K. M. Lemberg, M. R. Lamprecht, R. Skouta, E. M. Zaitsev, C. E. Gleason, D. N. Patel, A. J. Bauer, A. M. Cantley, W. S. Yang, B. Morrison, B. R. Stockwell, Ferroptosis: An iron-dependent form of nonapoptotic cell death. *Cell* **149**, 1060–1072 (2012).

58. B. R. Stockwell, J. P. F. Angeli, H. Bayir, A. I. Bush, M. Conrad, S. J. Dixon, S. Fulda, S. Gascón, S. K. Hatzios, V. E. Kagan, K. Noel, X. Jiang, A. Linkermann, M. E. Murphy, M. Overholtzer, A. Oyagi, G. C. Pagnussat, J. Park, Q. Ran, C. S. Rosenfeld, K. Salnikow, D. Tang,

F. M. Torti, S. V. Torti, S. Toyokuni, K. A. Woerpel, D. D. Zhang, Ferroptosis: A regulated cell death nexus linking metabolism, redox biology, and disease. *Cell* **171**, 273–285 (2017).

59. E. P. Amaral, D. L. Costa, S. Namasivayam, N. Riteau, O. Kamenyeva, L. Mittereder, K. D. Mayer-Barber, B. B. Andrade, A. Sher, A major role for ferroptosis in Mycobacterium tuberculosis–induced cell death and tissue necrosis. *J. Exp. Med.* **216**, 556–570 (2019).

60. H. H. Dar, Y. Y. Tyurina, K. Mikulska-Ruminska, I. Shrivastava, H.-C. Ting, V. A. Tyurin, J. Krieger, C. M. S. Croix, S. Watkins, E. Bayir, G. Mao, C. R. Armbruster, A. Kapralov, H. Wang, M. R. Parsek, T. S. Anthonymuthu, A. F. Ogunsola, B. A. Flitter, C. J. Freedman, J. R. Gaston, T. R. Holman, J. M. Pilewski, J. S. Greenberger, R. K. Mallampalli, Y. Doi, J. S. Lee, I. Bahar, J. M. Bomberger, H. Bayır, V. E. Kagan, *Pseudomonas aeruginosa* utilizes host polyunsaturated phosphatidylethanolamines to trigger theft-ferroptosis in bronchial epithelium. *J. Clin. Invest.* **128**, 4639–4653 (2019).

61. L. Qiang, Y. Zhang, Z. Lei, Z. Lu, S. Tan, P. Ge, Q. Chai, M. Zhao, X. Zhang, B. Li, Y. Pang, L. Zhang, C. H. Liu, J. Wang, A mycobacterial effector promotes ferroptosis-dependent pathogenicity and dissemination. *Nat. Commun.* **14**, 1430 (2023).

62. Y.-S. Lee, D.-H. Lee, H. A. Choudry, D. L. Bartlett, Y. J. Lee, Ferroptosis-induced endoplasmic reticulum stress: Cross-talk between ferroptosis and apoptosis. *Mol. Cancer Res.* **16**, 1073–1076 (2018).

63. Y. Zheng, L. Sun, J. Guo, J. Ma, The crosstalk between ferroptosis and anti-tumor immunity in the tumor microenvironment: Molecular mechanisms and therapeutic controversy. *Cancer Commun.* **43**, 1071–1096 (2023).

64. Y. Xie, W. Hou, X. Song, Y. Yu, J. Huang, X. Sun, R. Kang, D. Tang, Ferroptosis: Process and function. *Cell Death Differ.* **23**, 369–379 (2016).

65. S. Matsuzaki, T. Hiratsuka, M. Taniguchi, K. Shingaki, T. Kubo, K. Kiya, T. Fujiwara, S. Kanazawa, R. Kanematsu, T. Maeda, H. Takamura, K. Yamada, K. Miyoshi, K. Hosokawa, M.

Tohyama, T. Katayama, Physiological ER stress mediates the differentiation of fibroblasts. *PLOS ONE* **10**, e0123578 (2015).

66. Q. Yu, B. Zhao, J. Gui, K. V. Katlinski, A. Brice, Y. Gao, C. Li, J. A. Kushner, C. Koumenis, J. A. Diehl, S. Y. Fuchs, Type I interferons mediate pancreatic toxicities of PERK inhibition. *Proc. Natl. Acad. Sci. U.S.A.* **112**, 15420–15425 (2015).

67. R: The R Project for Statistical Computing. www.r-project.org/.

68. Y. Hao, S. Hao, E. Andersen-Nissen, W. M. Mauck, S. Zheng, A. Butler, M. J. Lee, A. J. Wilk, C. Darby, M. Zager, P. Hoffman, M. Stoeckius, E. Papalexi, E. P. Mimitou, J. Jain, A. Srivastava, T. Stuart, L. M. Fleming, B. Yeung, A. J. Rogers, J. M. McElrath, C. A. Blish, R. Gottardo, P. Smibert, R. Satija, Integrated analysis of multimodal single-cell data. *Cell* **184**, 3573–3587.e29 (2021).

69. A. Butler, P. Hoffman, P. Smibert, E. Papalexi, R. Satija, Integrating single-cell transcriptomic data across different conditions, technologies, and species. *Nat. Biotechnol.* **36**, 411–420 (2018).

70. R. Satija, J. A. Farrell, D. Gennert, A. F. Schier, A. Regev, Spatial reconstruction of single-cell gene expression data. *Nat. Biotechnol.* **33**, 495–502 (2015).

71. T. Stuart, A. Butler, P. Hoffman, C. Hafemeister, E. Papalexi, W. M. Mauck, Y. Hao, M. Stoeckius, P. Smibert, R. Satija, Comprehensive integration of single-cell data. *Cell* **177**, 1888–1902.e21 (2019).

72. B. Wong, Points of view: Color blindness. *Nat. Methods* **8**, 441–441 (2011).

73. I. Korsunsky, A. Nathan, N. Millard, S. Raychaudhuri, Presto scales Wilcoxon and auROC analyses to millions of observations. bioRxiv 653253 [Preprint] (2019). https://doi.org/10.1101/653253.

74. A. Subramanian, P. Tamayo, V. K. Mootha, S. Mukherjee, B. L. Ebert, M. A. Gillette, A. Paulovich, S. L. Pomeroy, T. R. Golub, E. S. Lander, J. P. Mesirov, Gene set enrichment

analysis: A knowledge-based approach for interpreting genome-wide expression profiles. *Proc. Natl. Acad. Sci. U.S.A.* **102**, 15545–15550 (2005).

75. G. Korotkevich, V. Sukhov, N. Budin, B. Shpak, M. N. Artyomov, A. Sergushichev, Fast gene set enrichment analysis. bioRxiv 060012 [Preprint] (2021). https://doi.org/10.1101/060012.

76. Y. Benjamini, Y. Hochberg, Controlling the false discovery rate: A practical and powerful approach to multiple testing. *J. R. Stat. Soc. B. Methodol.* **57**, 289–300 (1995).

77. K. J. Livak, T. D. Schmittgen, Analysis of relative gene expression data using real-time quantitative PCR and the $2^{-\Delta\Delta CT}$ method. *Methods* **25**, 402–408 (2001).

78. R. A. G. da Silva, W. H. Tay, F. K. Ho, F. R. Tanoto, K. K. L. Chong, P. Y. Choo, A. Ludwig, K. A. Kline, *Enterococcus faecalis* alters endo-lysosomal trafficking to replicate and persist within mammalian cells. *PLOS Pathog.* **18**, e1010434 (2022).

79. C.-C. Liang, A. Y. Park, J.-L. Guan, In vitro scratch assay: A convenient and inexpensive method for analysis of cell migration in vitro. *Nat. Protoc.* **2**, 329–333 (2007).

80. N. S. Ariffin, The CellProfiler pipeline analysis of cell migration. *Acta Histochem.* **125**, 152074 (2023).

81. A. Suarez-Arnedo, F. Torres Figueroa, C. Clavijo, P. Arbeláez, J. C. Cruz, C. Muñoz-Camargo, An image J plugin for the high throughput image analysis of in vitro scratch wound healing assays. *PLOS ONE* **15**, e0232565 (2020).

82. C. Kristich, N. T. Le, A. Barnes, S. Grindle, G. Dunny, Development and use of an efficient system for random mariner transposon mutagenesis to identify novel genetic determinants of biofilm formation in the core *Enterococcus faecalis* genome. *Appl. Environ. Microbiol.* **74**, 3377–3386 (2008).

83. H. V. Nielsen, P. S. Guiton, K. A. Kline, G. C. Port, J. S. Pinkner, F. Neiers, S. Normark, B. Henriques-Normark, M. G. Caparon, S. J. Hultgren, The metal ion-dependent adhesion site motif

of the *Enterococcus faecalis* EbpA pilin mediates pilus function in catheter-associated urinary tract infection. *mBio* ***3***, e00177-12 (2012).

84. G. M. Dunny, B. L. Brown, D. B. Clewell, Induced cell aggregation and mating in *Streptococcus faecalis*: Evidence for a bacterial sex pheromone. *Proc. Natl. Acad. Sci. U.S.A.* **75**, 3479–3483 (1978).

85. S.-B. Yoon, Y.-H. Park, S.-A. Choi, H.-J. Yang, P.-S. Jeong, J.-J. Cha, S. Lee, S. H. Lee, J.-H. Lee, B.-W. Sim, B.-S. Koo, S.-J. Park, Y. Lee, Y.-H. Kim, J. J. Hong, J.-S. Kim, Y. B. Jin, J.-W. Huh, S.-R. Lee, B.-S. Song, S.-U. Kim, Real-time PCR quantification of spliced X-box binding protein 1 (XBP1) using a universal primer method. *PLOS ONE* **14**, e0219978 (2019).
